# Healthcare professionals' views on psychological support for children and families affected by skin conditions in the UK: A qualitative study

**DOI:** 10.1002/ski2.376

**Published:** 2024-03-28

**Authors:** Olivia Hughes, Katherine H. Shelton, Andrew R. Thompson

**Affiliations:** ^1^ School of Psychology Cardiff University Cardiff Wales UK; ^2^ Doctoral Programme in Clinical Psychology Cardiff & Vale University Health Board & School of Psychology Cardiff University Cardiff Wales UK

## Abstract

**Background:**

Skin conditions can have a psychological impact on a child and their caregivers, however, support is not equally distributed between geographical regions in the United Kingdom (UK).

**Objectives:**

This study aimed to investigate the experience of National Health Service (NHS) healthcare professionals (HCPs) of addressing the psychological needs of children with skin conditions and their families, and gain expert opinion as to how services need to further develop.

**Design:**

HCPs were recruited to a qualitative study with an advert posted on social media.

**Methods:**

Fifteen HCPs took part in interviews, including dermatologists (*n* = 4), dermatology nurse consultants/specialists (*n* = 4), clinical psychologists (*n* = 4), liaison psychiatrists (*n* = 2), and a children's psychological well‐being practitioner (*n* = 1).

**Results:**

Thematic analysis revealed children often presented with anxiety, depression, self‐harm and suicidal ideation. The impact on caregivers was equally profound. There were differences in service provision across the UK and all HCPs recognised the urgent need for psychological support to be integrated into standard care. Participants described how a range of interventions are typically required including cognitive behavioural therapy (CBT), and systemic approaches, as well as mindfulness/third‐wave approaches. Barriers to the delivery of psychological services were associated with a lack of funding and training opportunities for core and specialist staff alike. However, in some instances, participants had overcome these challenges to be able to deliver unique services.

**Conclusions:**

There exist several barriers to providing paediatric psychological interventions, and many locations across the UK remain vulnerable as a result of continuing lack of national guidelines for the provision of psychological services.

**What is already known about this topic?**

- Psychological services for children with skin conditions are in short supply and not equally distributed across the United Kingdom.
- Both children and carers/parents can encounter a significant psychological burden associated with managing a skin disease, and the related social impacts of these conditions.
- Gathering information from expert clinicians is crucial in informing the delivery of high quality care.

**What does this study add?**

- This study adds to the literature illustrating the wide‐ranging psychological impact of paediatric skin conditions.
- The study highlights current barriers to service delivery in the United Kingdom, including funding/commissioning issues, a lack of training, and a lack of clear referral pathways into existing children and families mental health services.
- Healthcare professionals report the need for the greater provision of psychological support, and recommend further development and testing of specific psychological interventions.

## 1 INTRODUCTION

Childhood skin conditions can be emotionally challenging^1^, ^2^ and families must be provided with the appropriate level of support to buffer against the negative psychological impact. In delivering specialised services, it is not only the families of children with skin conditions who should be involved in design. As well as consulting patients,^2^, ^3^, ^4^ healthcare professionals (HCPs) are central to the process of ensuring an intervention meets target user needs and promotes behavioural change.^5^, ^6^ Co‐design with expert clinicians can facilitate an information gathering process by providing insights on what to prioritise or highlight anticipated barriers to delivery.^7^, ^8^

A range of therapeutic approaches have been applied to adults with skin conditions, including cognitive behavioural therapy (CBT),^9^ behavioural approaches (e.g., habit reversal) and stress/arousal reduction.^10^ There is also some evidence that educational approaches are being used within paediatric dermatology services.^11^ As well as these, evidence is increasing for the use of specific forms of CBT that integrate self‐compassion^12^ and mindfulness into the existing CBT paradigm.^13^ Specifically, mindfulness has shown promise for reducing anxiety and depression in children and adolescents living with physical health conditions^14^ and has been previously applied to a range of skin diseases.^15^, ^16^, ^17^, ^18^, ^19^, ^20^

Mindfulness involves cultivating an attitude of non‐judgement and non‐reactivity to experiences by learning to focus attention on the present moment.^21^ For people with skin conditions, practicing mindfulness could reduce physiological arousal (e.g., distress from negative reactions) which may be particularly relevant for dermatological symptoms that can be exacerbated by stress.^22^, ^23^ Indeed, the approach could be usefully applied to children and adolescents who may be experiencing heightened distress from an altered appearance affecting self‐confidence during an important developmental period.^24^ As well as this, mindfulness could improve relational functioning within families by creating an opportunity for bonding and promoting a sense of calm.^14^, ^25^ For example, mindful parenting has been found to reduce stress in caregivers of children with psoriasis and eczema.^26^ However, there is a lack of evidence for the use of psychological interventions with children with skin conditions, and further testing is required to ascertain the effectiveness of mindfulness and identify the underlying therapeutic mechanisms that warrant targeting.^2^, ^23^ Alongside this gap in the literature, there also appears to be a lack of agreement surrounding the psychological assessment of young people with skin conditions, pointing toward the need for further dermatology‐specific screening tools.^27^

To establish the importance of considering the mental health of paediatric patients and referral to specialist services, gathering data from dermatologists could be essential. For example, findings from a qualitative online survey of individuals in the United Kingdom (UK) with skin conditions showed that respondents felt there was a lack of support, and their needs and issues were not always fully understood by other people (including clinicians) suggesting that greater awareness, and provision of services is needed.^28^ To plan for service delivery, the involvement of dermatologists and other professionals with expertise in dermatology is crucial. A range of HCPs are involved in delivering psychological support to children and families and this is essentially central to the holistic care provided by dermatology nurses.

The delivery of psychological interventions or/and therapies is usually the role of psychological practitioners such as clinical, health, and counselling psychologists as well as professionals trained in designated therapies such as CBT. The inclusion of expert psychological practitioners with experience working with families affected by skin conditions could yield valuable information on how their mental health is managed, to increase understanding of what is important to prioritise when delivering interventions. Along with this, gathering psychologists' insights into running interventions will highlight the feasibility and acceptability of offering a range of approaches to support the well‐being of children and their parents.

Most recently, the British Society for Pediatric and Adolescent Dermatology have provided recommendations for the management of children with skin conditions, including improving assessment and adopting a psychosocial approach to care.^29^ Given the acknowledged need to increase psychological services within paediatric dermatology,^2^, ^28^ this study aimed to: (1) investigate the experience of HCPs in the National Health Service (NHS) of addressing the psychological needs of children with skin conditions and their families, and (2) gain expert opinion on the relevance and need for specific forms of psychological support. Importantly, this research builds on previous work by Hughes et al.^2^ investigating the parent and child experience of skin conditions and sought to gather evidence from HCPs to inform future interventions.

## 2 MATERIALS AND METHOD

### 2.1 Design

This study was a qualitative investigation using a mixed inductive‐deductive thematic analysis approach,^30^ that acknowledged some of the existing issues in service delivery (e.g., the unequal distribution of across the UK) whilst being open to capturing new/novel information (e.g., surrounding clinical management, views on therapeutic approaches, and foreseen barriers). Qualitative research methods are recommended by the Medical Research Council^31^ for the development of complex health interventions. When applied to dermatology, in‐depth data can be gathered surrounding peoples' experiences of the structure of services and adherence to treatment to identify training requirements.^32^, ^33^ For these reasons, adopting a qualitative approach is important for informing patient management by shaping the nature of new interventions.^32^, ^33^

### 2.2 Participants

Fifteen HCPs participated in semi‐structured interviews (Table 1). The sample size was sufficient for thematic analysis,^34^ and data was regularly assessed in terms of achieving saturation,^35^, ^36^ with consideration of gaining sufficient information power to address the research aims.^35^, ^37^

**TABLE 1:** Participant characteristics (*n* = 15).

Characteristic	Number
Gender	
Female	11
Male	4
Job role	
Dermatologist	4
Dermatology nurse consultant/specialist	4
Clinical psychologist	4
Liaison psychiatrist	2
Children's psychological well‐being practitioner	1
Age (years)	
30–40	5
40–50	2
50–60	6
60–70	2
Ethnicity	
White British	11
White British German	1
White German	1
Indian	1
White Irish	1
Geographic location	
England, UK	13
Wales, UK	2

### 2.3 Recruitment

Ethical approval was granted by Cardiff University (EC.22.09.20.6617R). To be included, participants were required to be healthcare professionals (e.g., practitioner psychologists, liaison psychiatrists, primary care psychological workers, dermatologists, specialist dermatology nurses) with experience working with children affected by skin conditions, in the UK, over 18 years, and an English speaker. Participants were recruited via an advertisement on social media and with direct email invitations.

### 2.4 Semi‐structured interviews

After informed consent was taken from each participant, a date for interview was agreed. Interviews were held online, and duration ranged from 44 to 87 min (mean = 65.66, median = 63 min). A flexible interview schedule was developed (Table 2), and HCPs were asked to anonymise clinical examples they spoke about. With permission, all interviews were video and audio recorded. Upon completion, participants were debriefed and discussions were transcribed verbatim.

**TABLE 2:** Example interview questions.

Question
Could you tell me about the psychological issues associated with having a skin condition that you see in children?
What are the implications for parent/carers and families of children with skin conditions?
Can you tell me about the psychological services that you are aware of for children and families?
What are your thoughts on offering a mindfulness‐based intervention involving exercises such as slow breathing, meditation, or focussing attention on one thing in the mind, to children with skin conditions and their families?
What do you think are the issues surrounding current available support options for children with skin conditions and their families?

### 2.5 Thematic analysis

Data were analysed with thematic analysis.^30^ O.H led the analysis using NVivo 12^38^ and began by reading and annotating transcripts. Key phrases were highlighted and labelled, followed by systematic coding, and grouping into main themes/subthemes. Themes were cross‐referenced with raw data in an iterative process to uphold rigour. To ensure interpretations were accurate, a member validation process^39^ was carried out, whereby participants were offered the opportunity to review their excerpts.

## 3 RESULTS

Thematic analysis led to the development of four themes and ten subthemes (see Table 3).

**TABLE 3:** Main themes and subthemes with supporting quotes from participant interviews.

**Theme 1: Psychological impact of skin conditions**
**1.1. Implications for children and adolescents:**
*“Children describe a really clear link between their emotional state and itch scratch cycles…it becomes more difficult to manage on a day when they're feeling anxious or stressed” (clinical psychologist).*
*“It's a really crucial age as adolescence you're going through a period of immense changes with your identity and the way your brain changes, and you're hardwired to be super sensitive to peer judgement, and you want to fit in” (clinical psychologist).*
*“The acne patients, I've had quite a few who from a psychological point of view, their acne has caused massive problems with their mood, suicidal thoughts, and self‐harming, just because they don't want to look the way that they look” (dermatology nurse consultant/specialist).*
*“Some of the anxiety and the depression is potentially driven by systemic, inflammatory process that might be happening in the in the brain, but we don't obviously know that for sure” (dermatologist).*
**1.2. Shared burden for parents and the family unit:**
*“Parents often just feel really sad that their child has this skin condition and that it's having a negative impact on them…there's maybe a bit of a process of grieving what they hoped life might be like for their child, and coming to terms with some of the ways that might look different” (clinical psychologist).*
*“Siblings may be affected if a lot of parental attention is needed and spent with creaming, bathing, oiling, hospital appointments and special skin care. Siblings may feel like losing out on parental 1:1 time and seek it directly or indirectly, presenting with demands on parents, sometimes with aches and pains and possible psychosomatic complaints” (liaison psychiatrist).*
*“Parents can feel mostly they want to help, and mostly they want to make things better, they can feel quite guilty sometimes…if it's an inherited condition” (dermatologist).*
*“Worry about fragility of the baby around damaging their skin and hurting them or causing pain…when a young person says ‘no, I don't want it’ or ‘no, it hurts’, it's really challenging for parents to hold in their mind that the child needs the treatment” (clinical psychologist).*
*“We've seen parents who've had to change their job because of the impact of not being able to sleep properly themselves, and the demands that the toddler is placing on them” (dermatologist).*
**1.3. Dynamics between parent and child:**
*“When a parent is stressed…it causes a flare up in the child's skin condition, and we know that there's a direct biological link between the skin and stress, and it happens both ways, when the skin has flared, that causes stress with the child, and the parent” (clinical psychologist).*
*“You've got relationship breakdowns because there may be differences in what the mum and dad think, you've got the sibling joining in because they're not getting any attention…that kind of arguments carrying on” (dermatology nurse consultant/specialist).*
*“Parents who've been much more bothered about the alopecia or the vitiligo than young person…allowing them to be okay is something that's really important for parents to be able to do, and not feel that they're somehow substandard because they've got a skin condition” (dermatologist).*
*“Where the parents are very supportive and they talk very openly about skin conditions, that it's just another thing, it's not a big deal, those children are much more malleable” (clinical psychologist).*
*“We don't ask the parents enough about ‘what do you do to cope with your child's eczema?’, ‘What do you do to look after yourself?’ We tend to focus on the child” (dermatologist).*
**Theme 2: Identifying distress in families**
**2.1. Initial assessment and approach to treatment:**
*“The way they're sitting, the way they're acting, the way they're behaving…but sometimes the clues are more subtle, things like they're behind in their milestones” (dermatology nurse consultant/specialist).*
*“You have to be frank, if you're talking about suicide, you use the suicide word, if you feel a child or adolescent is at risk of suicide, then you go more into the conversation of ‘are you a stage where you are going to go home tonight and commit suicide?’ You have to be really honest…you can't cover up the cracks” (dermatology nurse consultant/specialist).*
*“We would look at whether low mood is the main result or whether it's anxiety based, and then we'd offer the intervention that's relevant or a mixture of both, we often find that the children with anxiety have got low mood as well, because they're withdrawing”(children's psychological well‐being practitioner).*
*“[Family] communication can be quite shut down, especially when there's a daily treatment…the inclination is it's just so difficult that once it's over, just to push it away until they have to do it again, so there's kind of relentless cycle where they go from one day to the next…just having space as a whole family to just sit and look at that together with someone else can offer a lot” (clinical psychologist).*
*“I tend to see the child separately from the parents or guardian and then see the guardian separately from the child and then see them all together, so I try and pick up on any concerns” (dermatologist).*
**2.2. Standardised measures versus questions:**
*“Quality of life screening tools can be quite useful…a starting point for talking about different areas that are going well and areas that might be more difficult” (clinical psychologist).*
*“The best way to find out if there is distress is to ask open ended Socratic questions, so just say ‘how are you?’, ‘How is the family doing?’, ‘What impact does this have on the family?’ (dermatologist).*
*“Through practice and the work that's being done, the consensus is that it’s really about the history, there isn't a kind of scoring system that identifies distress, because people can anticipate the score low or high, it's a combination of their physical demeanour, which starts in the waiting room, what they look like when they come in, how interactive they are, we do generally do a DLQI, but that's not a score of emotional or psychological distress, but it might give you pointers” (dermatologist).*
*“I don't think that we're as good as we should be about the psychological impact of skin disease, we recognize it, but from a clinical or time point of view, we unfortunately don't spend enough time on it…we probably could do with more tools to identify those psychological problems” (dermatology nurse consultant/specialist).*
**Theme 3: Level of support provision**
**3.1. Current available psychological therapies:**
*“Mindfulness is always one that you would mention, because people know about it…we did try it in another hospital and it was successful, we saw there was a big difference in the people that used it” (dermatology nurse consultant/specialist).*
*“I use ACT with older adolescents…where people are talking about their skin getting in the way of them being able to do the stuff that matters to them, because that is kind of really the focus of ACT, identifying what is important to you and finding ways of moving towards that, despite the challenges you're facing” (clinical psychologist).*
*“CBT…the programme for low mood is behavioral activation, which is about doing more of what brings you happiness…and then the anxiety treatment is exposure work, so exposing you to the situations that you might be avoiding…and then we also do cognitive restructuring…we would challenge those thoughts to think about different ways to think about and manage their skin condition” (children's psychological well‐being practitioner).*
*“It's helpful to include families in some capacity in the treatment, if not to have a whole family focus because it can locate the problem in the young person, and in the young person's skin…and the solution in the child feeling differently about it, and therefore the behaviour is better, and the family is better” (clinical psychologist).*
*“You worry about how much you miss, it's not just that we don't have the time but we don't have the skills…we pick up on subtle cues but we are probably missing a lot of important signs” (dermatologist).*
**3.2. Lack of access to specialist services:**
*“I don't know of any specific psychological services, especially in this area…they've got fabulous psychodermatology services in different areas, but we don't have anything” (dermatology nurse consultant/specialist).*
*“There's a big South/North divide, and the services get less and less as you go further up…there's only 10 psychodermatology centres in the country, and people are travelling from Scotland to go to London” (dermatology nurse consultant/specialist).*
*“We've never had good psychological support in all the places I've worked, there aren't enough psychologists around who understand skin” (dermatology nurse consultant/specialist).*
*“The health board or trust just don't have that money or the staff to set up such a service” (dermatology nurse consultant/specialist).*
*“Access to support services for young people and families is really challenging at the moment, the waiting lists are huge” (dermatologist).*
**3.3. Healthcare professional recommendations for improving care:**
*“[Psychological support] is part of seeing someone, it's almost like giving another medication, it's just a fundamental part of holistic care for a problem that's not just in the skin but in the mind as well” (dermatologist).*
*“Having a direct [psychology] link would be ideal…specific to skin disease rather than just general mental health services…we're just a little bit behind in Wales” (dermatology nurse consultant/specialist).*
*“Psychological support should be standard…every single paediatric dermatology department needs to have access to inhouse psychology” (clinical psychologist).*
*“Healing the mind will have a very positive impact on the skin…with these severe inflammatory skin conditions, we've got increasingly targeted tools available like the biologics or small molecules, but at the same time, we are still suppressing symptoms, and if you ignore a very important part of the skin inflammation…then you're not realistically treating the patient and the stress caused by the skin disease…you're depriving the patient from one very important element of their treatment” (dermatologist).*
**Theme 4: Relevance of a mindfulness‐based intervention**
**4.1. Potential benefits of mindfulness:**
*“Mindfulness makes sense in terms of reducing anxiety and reducing stress and finding different ways of coping with those emotions…in relation to the itch scratch cycle…young people really like mindfulness and the breathing exercises as an alternative behaviour when they notice they're feeling stressed, or they feel like they want to scratch” (clinical psychologist).*
*“We do mindfulness bubbles a lot for the little ones, we use bubble machines…within a skincare routine…whilst they're waiting for their cream to sink in…they breathe up and breathe down when the bubbles are moving up and down” (clinical psychologist).*
*“One parent…painted [a rainbow] on their kids wall, and they all used to sit down and do breathing together…they were all involved and the whole family calmed down…it helped them in every way, it wasn't just about the child's skin condition” (clinical psychologist).*
*“[Mindfulness] would be more beneficial for some of the parents to be able to take 5 minutes, where it's not just all about the skin disease” (dermatology nurse consultant/specialist).*
*“Embedding it in things that are already part of somebody's routine tends to work…most people who I've worked with who have a regular mindfulness practice have tagged it onto something that's already part of their routine” (clinical psychologist).*
**4.2. Barriers to engagement in mindfulness:**
*“A lot of comments that we get from parents when we recommend things…you might get ‘I don't have time to do that’, or ‘I have other things to do’, and they tend to be really on board for helping the child, but in terms of trying actual techniques themselves, there's a bit of resistance” (children's psychological well‐being practitioner).*
*“Things like itch, I wonder whether mindfulness might make it worse? If you focus in the moment, on your body and how it's feeling, it could actually be horrendous if you've got a lot of itch” (liaison psychiatrist).*
*“Mindfulness is quite accessible for quite highly educated, non‐socially‐deprived people…for some young people in very difficult circumstances, telling them to accept things that aren't acceptable can be quite tricky” (dermatologist).*
*“Young people have found it quite difficult to engage with mindfulness because they are busy, and their brain can't settle…our experience has been mixed in terms of people continuing, they might do it in clinic, but then they might not continue” (dermatologist).*
*“Mindfulness has become more mainstream…it can be helpful because people are like, ‘oh, I've already heard of that I'd be willing to give it a try’, or it means that people are coming with a lot of baggage or misconceptions about what it is…and that can be a barrier” (clinical psychologist).*

### 3.1 Psychological impact of skin conditions

The impact of a skin condition is “more than skin deep” (dermatology nurse consultant/specialist) and can affect the psychological well‐being of children and their families. HCPs observed how “the skin and the mind are intimately connected” (dermatologist) and must be addressed together.

#### 3.1.1 Implications for children and adolescents

There can be “huge impacts on the well‐being, the long‐term effects of young people's ability to integrate, socialize, be motivated, and every aspect of life”. This included disruptions to schooling from a lack of sleep and pain, which could mean that “academically, these children will not do as well” (dermatology nurse consultant/specialist). Indeed, children regularly experienced distress from having an altered appearance. In many cases, these feelings led to clinical levels of “anxiety, depression, suicidal ideation” (dermatologist), and self‐harm. However, it appeared that the level of psychological distress was not always related to severity, and some children with mild disease were equally distressed as children with severe skin conditions. Physiologically, dermatologists described how “we're still trying to understand the links between skin inflammation, itching, sleep disturbance and potential brain inflammation” (dermatologist). There was also an observed association between emotions and the “propagation or exacerbation” (dermatologist) of symptoms.

#### 3.1.2 Shared burden for parents and the family unit

As well as children, their parents experienced worry, sleep loss, and shared the burden of managing treatment. The demands of treatment routines often dominated family life and resulted in additional considerations and planning of daily activities. HCPs described how these disruptions “can lead to mood disturbances and affective disorders in other members of the family” (dermatologist). For example, parents experienced concern over treatment and side effects of drug therapies. This also included a feeling of sadness from having an unwell child. However, the impact extended to siblings, who may miss out on parental attention.

#### 3.1.3 Dynamics between parent and child

The pressures placed on parents can lead to “all sorts of consequences including family dysfunction, or even breakdowns of families” (dermatologist). Psychologists highlighted the way in which parents respond to their condition could influence how a child copes. In cases where intensive care routines are required, the burden of treatment can “cause a lot of ruptures in family life” (clinical psychologist). There can also be skewed boundaries leading to the development of “very complex parental relationships” (dermatologist). However, managing hereditary skin conditions could increase bonding, as parents may be able to empathise from their own experiences. Nevertheless, parents could equally respond in less helpful ways if they encounter feelings of guilt or manage their condition differently to their child. Ultimately, HCPs speculated how increased levels of parental stress could contribute to the severity of the child's skin condition.

### 3.2 Identifying distress in families

HCPs described how they would identify distress during consultations, including assessing mental health. HCPs reported what they look for in terms of body language/emotional cues, and how they would manage a child that required support from specialist services.

#### 3.2.1 Initial assessment and approach to treatment

HCPs often began consultations by validating mental health concerns and to “not hide away from it, because it's very difficult as a professional sometimes to go down this road” (dermatology nurse consultant/specialist). This involved acknowledging the psychological challenges associated with skin conditions. Indeed, risk assessment is critical in dermatology, and emphasis was placed on the need for open discussions with children who might be experiencing distress. There were several signs in terms of body language that indicated distress in paediatric patients, including posture in clinic, behaviour during appointments (e.g., tearfulness, or outbursts of emotion), and milestone attainment. In addressing mental health, there was a need to manage expectations with consideration of curability and limited support services. Psychologists described approaching treatment by assessing symptoms to determine which form of intervention would be most appropriate. As well as this, HCPs discussed the benefits of having a family‐focussed approach to treatment, although this depended on relational dynamics.

#### 3.2.2 Standardised measures versus questions

Clinicians are encouraged to use a validated measure to assess the impact of a skin condition on a patient's quality of life. Indeed, several standardised measures were used, including: The Children's Dermatology Life Quality Index (CDLQI)^40^; Dermatology Life Quality Index (DLQI)^41^; The Revised Child Anxiety and Depression Scale (RCADS)^42^; The Patient‐Oriented Eczema Measure (POEM)^43^; The Patient‐Oriented Scoring for Atopic Dermatitis (PO‐SCORAD)^44^; Hospital Anxiety and Depression Scale (HADS).^45^ Despite this, not all of the scales are appropriate for younger children, but illustrate how HCPs sought to use a range of validated tools. Importantly, most of the HCPs described preferring to assess mental health by asking direct questions alongside questionnaires, to open up a conversation. Many HCPs described how existing quality of life measures were not always the most accurate tools for determining levels of distress, and pointed to the need for training in broader psychosocial assessment skills. Thus, it was highlighted how there can sometimes be discrepancies between psychometric scores and how a child is coping in reality, as well as the need for further work to improve dermatology‐specific screening tools.

### 3.3 Level of support provision

#### 3.3.1 Current available psychological therapies

When discussing psychological therapies, there were differences between psychology and dermatology staff. All psychologists discussed the specific psychotherapeutic approaches they would draw upon to target distress, however, dermatologists all spoke of the discrepancies between severity and psychological impact. Contrastingly, psychologists were clear in their recommendations for which interventions they would use, but dermatologists described the challenges arising from a lack of clear referral pathways. However, in the first instance, all HCPs agreed on the value of signposting to charities to “help fill the gaps” (dermatology nurse consultant/specialist). Several of the HCPs had working links with specialised services and were able to refer children to receive treatment. Where available, several interventions were offered, including CBT, mentalization‐based therapy, compassion‐focused therapy, mindfulness, and acceptance and commitment‐based therapy (ACT). Psychologists often combined different techniques according to the child's preferences and used individual formulation based interventions, as well as other strategies including progressive muscle relaxation and habit reversal.

#### 3.3.2 Lack of access to specialist services

There is a “lack of provision” of specialist services (clinical psychologist), and access can be “like a postcode lottery” (clinical psychologist). All HCPs noted the unequal distribution of specialised psychological support services across the UK. The difficulties in patients' accessing specialist services was speculated to be a result of a combination of different factors, including workforce, as well as funding for commissioning. For HCPs without direct access to specialised services, they referred to child mental health services, however, there were often long waiting times. Additionally, the importance of having a tailored psychological support intervention was highlighted, to address the specific symptoms associated with skin conditions.

#### 3.3.3 Healthcare professional recommendations for improving care

HCPs provided recommendations for how paediatric care could be improved with better access to psychological services. Considering the psychological impact of a skin condition was seen as equally important in dermatological care as prescribing drug therapies. Having a multidisciplinary approach with involvement from a psychologist was described by one dermatologist as helping to “unlock certain things…they can get to completely different layers of people's psyche compared to what I can do in my consultation with the patient, and then feed that back to me…so I can understand that patient better”. Further, another dermatologist felt that although they were able to “facilitate basic, affirmative CBT in every consultation…more developments would need a bit more training” to improve the psychological well‐being of patients.

### 3.4 Relevance of a mindfulness‐based intervention

HCPs were asked for their views on offering children and their parents a mindfulness‐based intervention, and whether, from their experience, they thought that this approach might be acceptable. HCPs were asked to give their insights into the potential benefits and barriers of delivering mindfulness.

#### 3.4.1 Potential benefits of mindfulness

Mindfulness can “centre you, make you feel calmer about life, it can help with anxiety, it can help with repetitive thoughts” (dermatologist). Several HCPs described having previously used mindfulness with children/families and “generally, it's very positive” (dermatologist). It was also speculated how the approach could promote positive physiological changes in the body and improve the skin condition itself “although we don't know how the brain or the mind‐skin axes really work, there could be implications for reducing inflammation if you can calm your mind”. Despite this, several HCPs felt mindfulness may be more beneficial for parents, as the impact on caregivers is often overlooked. In terms of delivery, an online format could promote engagement, as “real time interventions give patients a very specific responsibility to attend” (dermatologist). HCPs also felt “the tenants of mindfulness are useful but maybe not in the very formal way” (dermatologist).

#### 3.4.2 Barriers to engagement in mindfulness

HCPs highlighted several anticipated challenges to delivering a mindfulness‐based intervention. This included how “you have to make the time for it, it doesn't work if you just do ten minutes once a week” and “for busy parents with lots of caring needs, making that time, they might struggle” (clinical psychologist). HCPs described that for some children, sitting with thoughts and body sensations in a non‐judgemental way could be difficult or even “intensify their experience of itchiness” (liaison psychiatrist). In terms of acceptability, several HCPs raised the potential impact of socio‐economic status on accessibility, and the need to ensure an intervention is equally available for all demographics of patients. Of note, one dermatologist spoke of how they had referred patients to try mindfulness and it had mixed reviews, suggesting that there could be individual preferences.

## 4 DISCUSSION

This study aimed to investigate the experience of HCPs of addressing the psychological needs of children with skin conditions and their families and gain expert opinion on specific forms of psychological intervention. Participants all described encountering a significant psychological burden in children,^2^, ^46^ including social withdrawal, interruptions to education, lack of sleep, and co‐morbid mental health conditions, such as anxiety, depression, and in severe cases, suicidal ideation and self‐harm. The burden was equally profound for parents (and siblings), who experienced disruptions to sleep, stress, and sadness from having an unwell child.^1^, ^47^

There was consensus for the need to conduct thorough assessments with a range of screening tools and skills to identify psychological distress (e.g., standardised measures, clinical observation, interviewing).^27^, ^29^ These findings are supportive of previous research carried out by the British Society for Pediatric and Adolescent Dermatology,^29^ and highlight how HCPs felt the most comprehensive way to assess distress was by asking direct questions, with assessment tools as a guide. Although this appeared to be the favoured approach, there could be restrictions in terms of time pressures during clinic appointments. As well as this, HCPs must feel confident enough to have open discussions with families about mental health. If clinicians are regularly asking about psychological well‐being and identifying problems, it is imperative there is somewhere to refer patients to. However, all HCPs felt support services were unequally distributed across the UK and were not meeting demand.^48^ The disparity in care was speculated to be for a range of reasons including a lack of: (1) mental health training, (2) funding, and (3) unclear referral pathways. HCPs made recommendations for how dermatology care could be improved for children and their families, with additional training, commissioning, and standardised access to psychological support.^28^, ^48^, ^49^

Despite this, many HCPs had overcome the challenges; trained psychologists typically discussed their experience of delivering a range of therapies (e.g., CBT, ACT, mindfulness, mentalization‐based therapy, compassion‐focused therapy, family/systemic therapy), whilst dermatology professionals described using simple single therapeutic techniques (e.g., habit reversal), and sometimes complimentary approaches were also offered (e.g., yoga, progressive muscle relaxation). Further, HCPs felt mindfulness could be helpful for children experiencing anxiety.^2^ Several dermatologists described how mindfulness could potentially reduce inflammation and upregulate healing processes. Although biological mechanisms were discussed by HCPs in a theoretical manner, there were anecdotal examples of stress being observed as a precipitating factor in the exacerbation of symptoms and maintenance of vicious itch/scratch cycles. Indeed, there is evidence to suggest that activation of the body's stress pathways (e.g., hypothalamic‐pituitary‐adrenal [HPA] axis) could impact on skin homeostasis.^50^ Activities such as meditation could reduce activation of the nervous system and mediate physiological reactivity to stress.^51^ However, the stress‐inflammation pathway is complex, and further research is required to test the efficacy of this approach and understand the underlying mechanisms.^52^

Mindfulness had been used by several clinical psychologists, who spoke of favourable outcomes for skin‐related symptoms. However, some HCPs who had experience of offering mindfulness‐based interventions described how not all patients had engaged with practices for a combination of reasons, including previously highlighted misconceptions surrounding the nature of mindfulness.^2^, ^53^ HCPs provided suggestions for how mindfulness could be delivered to families, including using an online format to promote adherence.^2^ Participants felt a more informal mindfulness curriculum might be easier for families to engage with, as they often had complex treatment routines which might limit how much time they have to practice an intensive intervention. Indeed, similar findings have been previously reported by parents of children with skin conditions themselves.^2^ Of note, several HCPs felt that mindfulness could be usefully applied to manage levels of parenting stress, and there is emerging evidence to support this.^26^ Importantly, both dermatologists and psychologists highlighted the importance of taking a systemic approach to consider a child in the context of their family environment, suggesting that alternative psychotherapeutic approaches also warrant investigation, such as family therapy.

However, this study does have several limitations. Whilst the sample size was appropriate for this design, participants were self‐selecting and had an interest in psychodermatology, which may affect transferability to general practice. As such, additional surveys might compliment this study by considering the extent to which these findings are mirrored in other services and in other countries. Further, this study focussed on Wales and England, and did not include participants from other UK nations (i.e., Northern Ireland or Scotland).

Overall, dermatology and psychology healthcare professionals openly recognise the shared burden experienced by families affected by skin conditions, but are often limited in providing psychological support. Although many clinicians across England were able to deliver specialised services to children and their parents, the provision was determined by location in the UK. There was consensus from all healthcare professionals in the need to integrate psychological services into the dermatological pathway of care as a national standard, to reduce the disparities in access to specialised care.

## CONFLICT OF INTEREST STATEMENT

O.H is a Patient Associate Editor for the British Journal of Dermatology. A.R.T is an Associate Editor for the British Journal of Dermatology and has received research support or/and honorarium in the last 12 months from Sanofi, Pfizer, and UCB. He is also a Trustee to the charity Changing Faces and a member of scientific advisor board of The Vitiligo Society.

## AUTHOR CONTRIBUTIONS

**Olivia Hughes**: Conceptualization (equal); data curation (lead); formal analysis (lead); investigation (lead); project administration (equal); writing – original draft (lead); writing – review & editing (lead). **Katherine H. Shelton**: Conceptualization (lead); methodology (lead); project administration (equal); supervision (lead); writing – original draft (supporting); writing – review & editing (equal). **Andrew R. Thompson**: Conceptualization (lead); methodology (lead); project administration (equal); supervision (lead); writing – original draft (supporting); writing – review & editing (equal).

## ETHICS STATEMENT

Ethical approval was granted by Cardiff University (EC.22.09.20.6617R).

## Data Availability

The data that support the findings of this study are available from the corresponding author upon reasonable request.
